# Integrating Metal-Oxide-Decorated CNT Networks with a CMOS Readout in a Gas Sensor

**DOI:** 10.3390/s120302582

**Published:** 2012-02-27

**Authors:** Hyunjoong Lee, Sanghoon Lee, Dai-Hong Kim, David Perello, Young June Park, Seong-Hyeon Hong, Minhee Yun, Suhwan Kim

**Affiliations:** 1 Department of Electrical Engineering, Seoul National University, Seoul, 151-744, Korea; E-Mails: hyunjoong.lee@amic.snu.ac.kr (H.L.); lov4holic@gmail.com (S.L.); ypark@snu.ac.kr (Y.J.P.); 2 Department of Materials Science and Engineering and Research Institute of Advanced Materials, Seoul National University, Seoul, 151-744, Korea; E-Mails: daihong@snu.ac.kr (D.-H.K.); shhong@snu.ac.kr (S.-H.H.); 3 Department of Electrical and Computer Engineering, Swanson School of Engineering, University of Pittsburgh, Pittsburgh, PA 15261, USA; E-Mails: dperello16@gmail.com (D.P.); miy16@pitt.edu (M.Y.)

**Keywords:** carbon nanotube (CNT), read-out integrated circuit, single die, metal decoration

## Abstract

We have implemented a tin-oxide-decorated carbon nanotube (CNT) network gas sensor system on a single die. We have also demonstrated the deposition of metallic tin on the CNT network, its subsequent oxidation in air, and the improvement of the lifetime of the sensors. The fabricated array of CNT sensors contains 128 sensor cells for added redundancy and increased accuracy. The read-out integrated circuit (ROIC) was combined with coarse and fine time-to-digital converters to extend its resolution in a power-efficient way. The ROIC is fabricated using a 0.35 μm CMOS process, and the whole sensor system consumes 30 mA at 5 V. The sensor system was successfully tested in the detection of ammonia gas at elevated temperatures.

## 1. Introduction

The detection of chemicals is critical for processes such as industrial process control and environmental monitoring. Nanostructure sensors are small and consume little power, while also effectively detecting many chemicals in real time. Carbon nanotubes (CNT) are one promising candidate as an active element in such sensors because of high sensitivity resulting from large surface area. Carbon nanotubes (CNT) were initially characterized by Iijima [1] in 1991, and CNT-based chemical sensors were demonstrated in 2000 [2,3]. The special geometry of the CNT makes it electrochemically active. Further, the electrical properties of a CNT network to be modulated by various chemicals such as NH_3_ and NO_2_. The sensing mechanism of a CNT differs between reactants, but it is generally known that electron-accepting molecules (e.g., NO_2_) and electron-donating molecules (e.g., NH_3_) can interact with CNTs directly or indirectly and modify the Fermi level. The result is that a CNT sensor can have lower resistance and operating temperature than an equivalent metal-oxide sensor. However, there are limitations dependent upon the device structure including irreversibility, long recovery time, and insufficient selectivity.

The two elementary types of CNT gas sensors: single aligned CNT devices and the simpler random network CNT type. In the case of a single aligned CNT, the sensors offer good sensitivity at room temperature, but the fabrication costs are high and the sensors offer limited selectivity with an irreversible response [2]. In response, metal-decorated CNT random network sensors have been researched [4,5]. These devices are formed by covering or “decorating” metal particles on the surface of the CNTs to enhance sensitivity to certain gases compared with bare CNT networks. Furthermore, better selectivity can be obtained in sensors intended to detect natural gases because sensitivity depends strongly upon the type of metal deposited. Metal-decorated random-network CNT network sensors often display both better reversibility [4–8], and fabrication procedures are more easily scaled than the single-CNT type. Applying signal processing techniques such as pattern matching to the output of an array of metal-decorated CNT cells allows a multi-modal sensor to be constructed which is able to detect several different gases [9].

Nevertheless, metal-decorated random network sensors have a rather wide resistance range, unpredictable performance, and a limited selectivity. These difficulties can be overcome with a multi-cell CNT network structure, which averages out single-device variations [9]. A recording system for this type of sensor has also been developed [10], but it is not appropriate for ubiquitous sensor networks because the CNT sensors and the read-out circuit are not integrated onto a single die. Furthermore, the bare CNT networks in the previous work suffer from a poor reversibility and a short lifetime.

Therefore, in this work we combine metal-oxide-decorated CNT network gas sensors with a read-out circuit on a single die to produce a system suitable for ubiquitous sensor network applications. The chemistry that we will consider in this paper is restricted to the detection of NH_3_ gas using SnO_2_-decorated CNT networks. This scenario is sufficient to allow us to verify the operation of our sensor system, but its chemistry can easily be extended to many other gases.

In Section 2 of this paper we introduce the fabrication of CNT network sensors, compare bare CNT networks with metal-decorated CNT networks, and mention the measurement system. In Section 3 we introduce our single-die, multi-cell sensor architecture which includes a read-out circuit. Circuit design issues and the overall structure of the sensor system are all discussed in-depth. Section 4 covers the measured results and discussion topics related to this sensor system and we make some concluding remarks in Section 5.

## 2. Experimental: Carbon Nanotube Sensing Cell Fabrication

**Figure 1.**
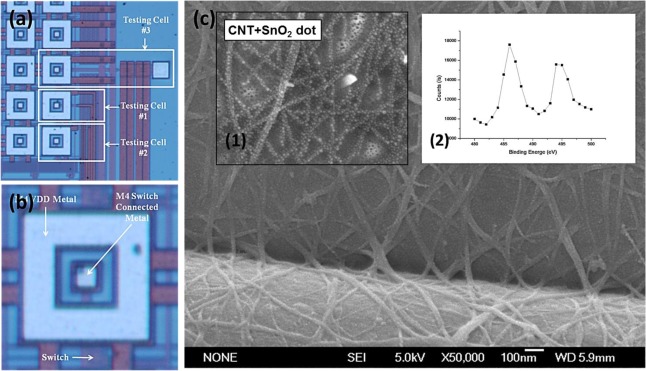
CNT sensing cell information. (**a**) Optical image of CNT test cells. (**b**) Individual CNT cell. (**c**) SEM image of the SnO_2_-decorated CNT network. (**c.1**) SEM image of the SnO_2_-decorated CNT network at higher magnification. (**c.2**) Core-level XPS Sn3d spectrum indicating oxidation of the Sn.

To allow an array of metal-oxide-decorated CNT network sensors to share a die with an ROIC, a concentric electrode were fabricated on the die using conventional complementary metal oxide semiconductor (CMOS) processes, as shown in Figure 1(a,b). The enclosing and island electrodes of each cell were constructed from a top metal layer (M4, Al), and the oxide layer applied during die passivation was partially eliminated from both electrodes to interface the sensors to the ROIC. Then the aluminum electrodes were gold-plated to reduce the contact resistance between electrodes and the CNT network sensors. Platinum plating is generally considered more suitable for gas sensing applications in which platinum acts as a catalyst, but gold-plated electrodes are more suitable for biological sensing applications, as reported in previous research [11]. To construct the CNT networks between the inner and outer electrodes, p-type single-walled CNTs (0.05 g) were immersed in a 1 L bath of 1,2-dichlorobenzene, and the die was dipped into that solution [12,13]. To avoid shorting the contact pads, the die was only immersed deeply enough to deposit the CNTs on the electrodes.

Subsequently, SnO_2_ nanoparticles were deposited by thermal evaporation. The deposition was performed with a room-temperature chuck to avoid damage to the other CMOS devices on the die. The CNT-loaded CMOS circuits were attached to a 4 inch silicon wafer and put into an MH-1800 thermal evaporator, using 99.999% Sn as the metal source. Deposition was performed at a rate of 0.1 Å/s for 100 s, at a pressure of 7.0 × 10^−6^ torr. After deposition, the die was extracted from the chamber, and the metallic tin was naturally oxidized by air at room temperature.

Figure 1(c.1) and Figure 1(c.2) inset 1 show SEM images of the CNT network decorated with SnO_2_ nanoparticles. We can see that the CNTs have been homogeneously distributed between electrodes and connected into a network by the dip-coating process. The SnO_2_ nanoparticles deposited on the CNT networks by evaporation are spherical, with diameters of 3–5 nm. Because these dots of tin are so small, they are easy to oxidize. To check the extent of this oxidization, the chemical state of the SnO_2_ was examined using a Sigma Probe X-ray photoelectron spectroscope (from ThermoVG, UK) with Al K radiation (1,486.6 eV). Inset 2 of Figure 1(c) shows the core-level X-ray photoelectron spectroscopy (XPS) spectrum for Sn3d, which were calibrated to the hydrocarbon C1s line at 284.5 eV. The Sn3d spectrum exhibits a double feature at 494.6 eV (3d3/2) and 486.3 eV (3d5/2). These peaks agree respectively with the values for the tin and oxygen that comprise the lattice structure of SnO_2_ [14].

**Figure 2.**
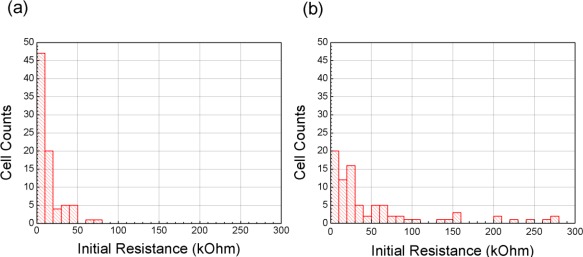
CNT network initial resistances. (**a**) Distribution of initial resistance for the bare CNT networks. (**b**) Distribution of initial resistance for the SnO_2_-decorated CNT networks.

Figure 2 shows the distribution of initial resistance for the bare CNT networks and the SnO_2_-decorated networks. We see that the initial resistances of individual sensors are increased by the decoration processes, and that they are also widely distributed. Fully determining the reasons for these changes is beyond the scope of this paper, but we speculate that two factors are involved: the depletion of charge in the CNTs and added charge centers after SnO_2_ deposition. CNTs are extremely sensitive to the addition of environmental charge centers, resulting in decreased current and trap-assisted conductivity [4–6,15–17]. Although CNT are remarkable resistant to damage via low-energy species [18], there remains the possibility of damage to the CNT networks during SnO_2_ deposition. These combined factors are likely to obstruct currents flowing across the CNT networks and increase overall resistance.

**Figure 3.**
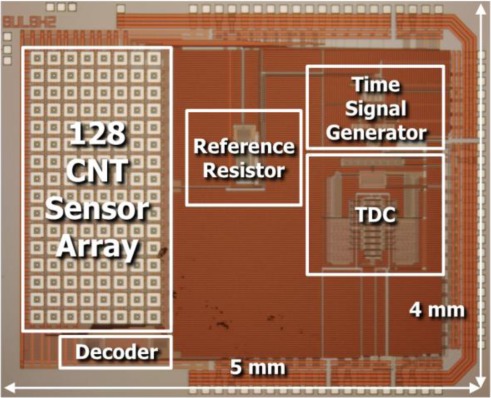
Optical image of the CNT ROIC die.

**Figure 4.**
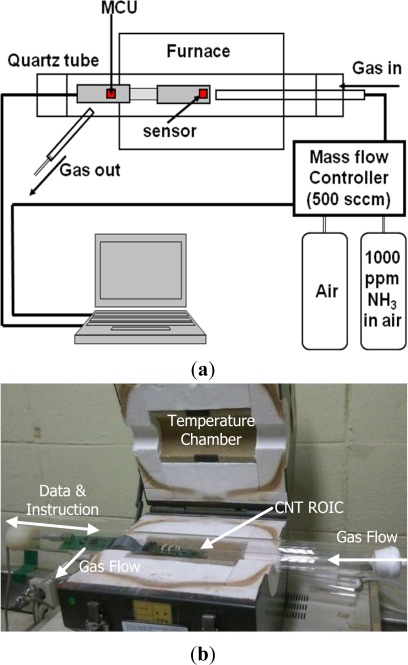
CNT sensor measurement system. (**a**) Schematic diagram showing the concept and operation of the temperature-controlled measurement system. (**b**) Photograph of the test equipment.

The CNT ROIC chip was fabricated using a 0.35 μm CMOS process, as shown in the photograph in Figure 3. Integrating the CNT networks and the ROIC on the same die is done by locating the CNT cells on the left-had side of the ROIC, while the pads are located on the right. With this layout, it is possible to dip the cells into the CNT solution as described above, while the keeping the pads above the liquid to avoid introducing shorts between them. The CNT ROIC die is then connected to the PCB using the chip-on-board process, with the CNT sensor cells remaining exposed to react with gas. Figure 4 shows the arrangements for testing the CNT ROIC. The experimental sensing setup utilizes mass flow controllers interfaced to a computer and a 38 mm diameter quartz reaction tube. The circuit is powered via a USB connection to the computer, which serves as both a 5 V power supply and data interface. In this manner, air (21% O_2_/N_2_, 99.999%) with or without 1,000 ppm of NH_3_ gas can be introduced into a temperature-controlled environment while performing electrical characterization. This arrangement also promotes uniform flow across the array of CNT cells, which was fixed at 500 cm^3^/s.

## 3. Design and Theory

### 3.1. The ROIC Architecture

#### 3.1.1. The CNT Cell Array Structure

The CNT cell array has eight rows and 16 columns. There are three test cells, and the remaining 125 cells are connected to the read-out circuit core that measures their resistance. Figure 1(b) shows the structure of one of the non-test cells. The outer ring of metal is connected to VDD and the metal at the centers is connected to the read-out circuit core. The passivation layer which was applied to both of these metal areas has been partially eliminated, as discussed in the previous section, to allow the cell to be interfaced to the read-out integrated circuit (ROIC). This process is the same as that used to open pads.

Figure 1(a) shows the structure of each of the three test cells in the array. The first of these cells is used to check whether the CNTs have been deposited satisfactorily, and for testing the switch circuit. In this cell, the outer and inner metal areas are both connected to the pad and the resistance of the CNT network deposited on that metal can be measured using a multimeter. This provides an initial check on CNT functionality which is independent of the ROIC. The input, output, and control signal lines of the switch are also linked to the pad, allowing the switch circuit to be tested without difficulty.

In the second test cell, the inner metal area is connected to the input signal line of the switch, which has its output signal and control signal lines are connected to the pad. This allows cell selection operations to be tested, as well as providing further verification of CNT functionality.

The third test cell has the same structure as the second, except that the inner metal area is connected to the pad and to the input signal line of the switch. This arrangement can be used to calibrate the ROIC, using a technique which will be described later.

**Figure 5.**
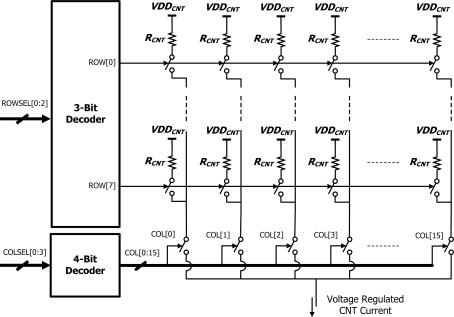
Circuit schematic for CNT sensor array. Individual CNT cells can be measured via the 3 bit row decoder and 4 bit column decoder.

Figure 5 shows the arrangement of the CNT sensor array circuit. A 7-bit digital code, ROWSEL[0:2] and COLSEL[0:3], assigned from outside the chip selects a single sensor cell. A 3-bit row decoder and a 4-bit column decoder respectively determines the row and column of the cell. When a CNT cell is connected to the ROIC, there exists a constant voltage drop across the CNT cell. This results from the voltage regulation loop inside the ROIC and produces a steady current through both cell and ROIC allowing the resistance of the CNT networks to be measured.

#### 3.1.2. Analog Front-End Circuit

There are two possible ways to read the resistance of the CNT network in a cell. We can either generate a constant current through the cell and measure the voltage across it, or apply a fixed voltage and measure the resulting current. The first method has good linearity because the resulting voltage is directly proportional to the resistance. But a complicated circuit is required to generate a constant current accurately. The second method requires us to deal with the inverse proportionality I = V/R, but the generation of a constant voltage is a much simpler task.

We use a simple circuit in which a constant voltage is applied to the CNT cell and the resulting current charges a capacitor. The charging time is measured until the voltage across this capacitor exceeds a threshold. This charging time can be expressed as follows:

(1) $$\Delta T=\frac{C\Delta V_{\text{Charge}}}{\text{VDD}-V_{\text{Regulation}}}\;R.$$

Indirect measurement of the current as a charging time allows accurate measurements to be made by a simple circuit. The simplest way of measuring the charging time of the capacitor is to count clock cycles. Then the dynamic range of the ROIC is only limited by the shortest and longest time that can be measured. The shortest detectable time is fixed by the clock period, but it can be extended by introducing a time-to-digital converter (TDC) circuit based on a delay-locked loop (DLL). This TDC circuit will be discussed in detail in the next section. The longest detectable time is determined by the number of bits in the clock cycle counter.

**Figure 6.**
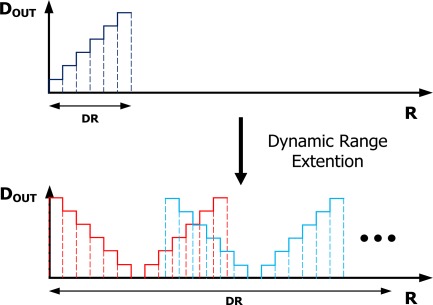
Dynamic range extension scheme to allow a greater range of charging times of an output capacitor to be measured. The improved timing ranges allow greater accuracy for simple and effective on-chip measurement of the current.

We use the dynamic range extension scheme shown in Figure 6 to increase the charging time that we can measure with a given range of detectable times. We introduce an on-chip switching reference resistor, a replica regulator and a replica time-signal generator, consisting of a comparator and a capacitor. The voltage drop across the cell and the on-chip reference resistor is the same, and each of the resulting currents charges an identical capacitor. The difference between the time required to charge these two capacitors is digitized by the read-out circuit. The resistance of on-chip reference resistor can be changed by a external digital code, which achieves a wide dynamic range.

**Figure 7.**
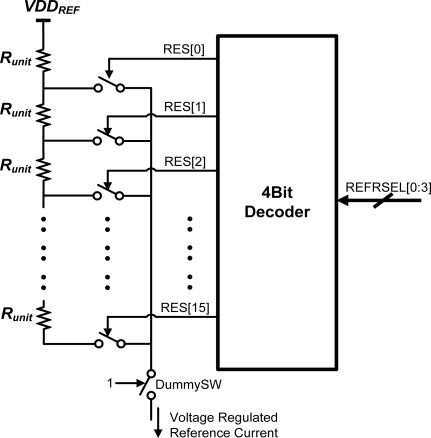
The reference resistor string. Each resistor RES[] is 20 kΩ, permitting the resistance to be dynamically varied from 20 kΩ to 320 kΩ in 20 kΩ steps.

**Figure 8.**
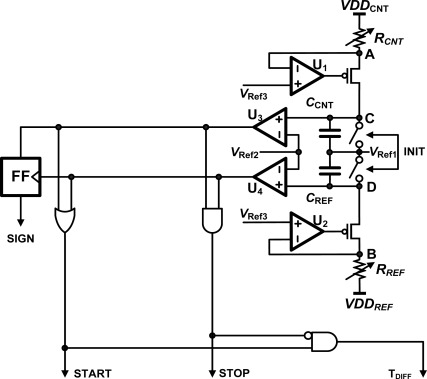
The analog-front-end circuit of the CNT ROIC used for measurement of the current across the CNT sensor cell.

Figure 7 shows the on-chip reference string of 20 kΩ resistors. Its overall resistance can be set from 20 kΩ to 320 kΩ, in steps of 20 kΩ, by an externally supplied 4-bit code, REFRSEL[0:3]. Figure 8 shows the analog front-end circuit of the CNT ROIC. An op-amp and a PMOS transistor maintain the voltage at nodes A and B at V_Ref3_. When the INIT signal is high, the capacitors C_REF_ and C_CNT_ are discharged and the voltage at nodes C and D becomes V_Ref1_. When the INIT signal goes low, two capacitors are charged and the voltages at nodes C and D increase, but the rates at which the voltages at C and D increase are different because the CNT network and the reference resistor can be expected to have different resistances. When the voltage at either node reaches V_Ref2_, the START signal goes high; and when the second node also reaches V_Ref2_, the STOP signal goes high. The T_DIFF_ signal which controls the clock cycle counter goes high with the START signal, and then goes low again when the STOP signal goes high. The clock cycle counter and the DLL-based TDC together convert the time of the between arrival of the START and STOP signals to a digital code. These circuits will be discussed at next section. The SIGN signal indicates whether node C or node D reached V_Ref2_ first. If it was C, then the SIGN signal is high, but if it was D the SIGN signal is low. This SIGN signal and the encoded 12-bit output data can be used by the program in a controller or PC to determine the resistance of the CNT network at a selected cell.

#### 3.1.3. DLL-Based TDC

The T_DIFF_ signal generated by the analog front-end circuit is converted to a digital code by the TDC. The simplest form of TDC is a clock cycle counter. When the T_DIFF_ signal goes high the clock cycle counter is enabled, and it then operates until the T_DIFF_ signal goes low. With this arrangement, the maximum measurable time range is determined by the total number of bits of the clock cycle counter. For our design, we selected 8 bits.

**Figure 9.**
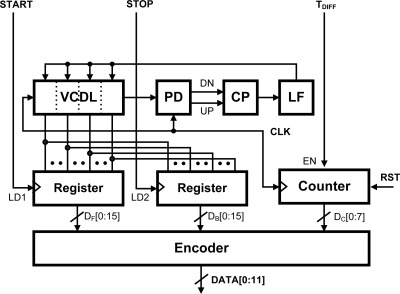
Analog to digital TDC circuit in the CNT ROIC.

**Figure 10.**
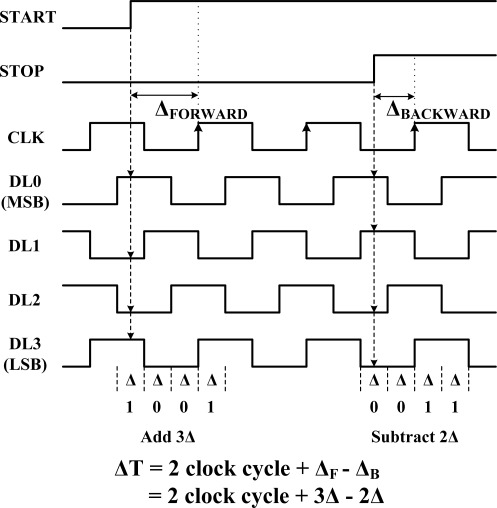
Example of fine TDC operation (with 4 delay cells). The real design as 16 delay cells.

The resolution of this type of TDC is determined by the length of a clock cycle. This resolution can be improved by multiplying the clock frequency, but power consumption is directly proportional to this frequency. Instead, we combine a DLL-based fine TDC with a counter-based coarse TDC as shown in Figure 9. A DLL is used to generate multi-phase clock signals in the fine TDC. Once the DLL is locked, assuming there is no harmonic lock problem, the DLL output clock is delayed by a single clock cycle with respect to the input clock. Thus the delay produced by each cell in the DLL is a clock period divided by the total number of delay cells. The START and STOP signals sample the clock phases and cause the states of the delay-line to be stored in registers. These states fix times of arrival of the START and STOP signals within the clock period. The sampled code is changed to binary, and then added to, or subtracted from, the clock cycle counter code. Figure 10 shows the operation of the fine TDC diagrammatically, using 4 delay cells. The actual design has 16 delay cells.

We have already described how a clock cycle counter is used to obtain a coarse time with a wide range. The delay-lines of the DLL obtain the fine time by interpolation within the period of the reference clock CLK. Thus the encoder receives three digital codes, D_COUNTER_ from the counter and D_FORWARD_ and D_BACKWARD_ from the delay-line registers. From this data it determines an n-bit digital code, D_OUT_, which is the digital representation of T_DIFF_ signal as follows:

(2) $$D_{\text{OUT}}=D_{\text{COUNTER}}\times 2^{M}+(D_{\text{FORWARD}}-D_{\text{BACKWORD}})$$

### 3.2. Calibration and Error Reduction

#### 3.2.1. Device Mismatch and Calibration

Although a circuit may be designed with matched sizes of MOS transistors or capacitors, they will not have truly identical dimensions in a real implementation because of technology limitations. This device mismatch effect is well known to cause problems such as op-amp offsets, and needs to be anticipated. In our analog front-end circuit, the charging times of nodes C and D can be expressed by the following equations:

(3) $${\Delta T}_{\text{CNT}}=\frac{R_{\text{CNT}}\times C_{\text{CNT}}\times \Delta {V^{\prime }}_{\text{CNT}}}{{\text{VDD}}_{\text{CNT}}-{V^{\prime }}_{\text{Ref},\text{CNT}}},\;\;\;\;{\Delta T}_{\text{REF}}=\frac{R_{\text{REF}}\times C_{\text{REF}}\times \Delta {V^{\prime }}_{\text{REF}}}{{\text{VDD}}_{\text{REF}}-{V^{\prime }}_{\text{Ref},\text{REF}}}$$

where V′_Ref,CNT_ = V_Ref3_ − V_OS1_, V′_Ref,REF_ = V_Ref3_ − V_OS2_, ΔV′_CNT_ = V_Ref2_ + V_OS3_ − V_Ref1_, and ΔV′_REF_ = V_Ref2_ + V_OS4_ − V_Ref1_. The four voltages V_OS_ are the offsets of the op-amps U_1_, U_2_, U_3_ and U_4_. The digital code T_DIFF_ output by the ROIC can be related to ΔV_CNT_ and ΔV_REF_ as follows:

(4) $$T_{\text{DIFF}}={\Delta T}_{\text{CNT}}-{\Delta T}_{\text{REF}}$$

From these two equations, we can obtain R_CNT_ as follows:

(5-1) $$R_{\text{CNT}}=\left(\frac{{\text{VDD}}_{\text{CNT}}-{V^{\prime }}_{\text{Ref},\text{CNT}}}{C_{\text{CNT}}\times \Delta {V^{\prime }}_{\text{CNT}}}\right)\times \left(\frac{R_{\text{REF}}\times C_{\text{REF}}\times \Delta {V^{\prime }}_{\text{REF}}}{{\text{VDD}}_{\text{REF}}-{V^{\prime }}_{\text{Ref},\text{REF}}}+T_{\text{DIFF}}\right)$$

which we can rewrite as:

(5-2) $$R_{\text{CNT}}=E_{\text{MIS}}R_{\text{REF}}+T_{\text{DIFF}}\times \left(\frac{{\text{VDD}}_{\text{CNT}}-{V^{\prime }}_{\text{Ref},\text{CNT}}}{C_{\text{CNT}}\times \Delta {V^{\prime }}_{\text{CNT}}}\right)$$

where E_MIS_ is the error due to the mismatch between the CNT network and the on-chip reference part in the analog front-end circuit, and is expressed as follows:

(5-3) $$E_{\text{MIS}}=\left(\frac{{\text{VDD}}_{\text{CNT}}-{V^{\prime }}_{\text{Ref},\text{CNT}}}{{\text{VDD}}_{\text{REF}}-{V^{\prime }}_{\text{Ref},\text{REF}}}\right)\;\left(\frac{C_{\text{REF}}}{C_{\text{CNT}}}\right)\;\left(\frac{\Delta {V^{\prime }}_{\text{REF}}}{\Delta {V^{\prime }}_{\text{CNT}}}\right).$$

In the Equation (5-2), device mismatches cause errors in the values of both the slope and the offset. These errors cannot be predicted, and thus the raw value of the digital output code of the ROIC need to be calibrated to obtain correct values of R_CNT_. Test cell #3, which we described earlier, it used for this calibration which is performed as follows: First, we select the row and column selection digital codes to select the test cell #3. Second, we set the value of R_REF_ by selecting the code that corresponds to test cell #3. Third, we set R_CNT_ by connecting an external resistor of the appropriate value between the ROIC core and the inner metal region of test cell #3. Fourth, we operate the ROIC and record the digital code T_DIFF_. By repeating these steps, a table relating T_DIFF_ to R_CNT_ can be obtained. This table can subsequently be used to calibrate the ROIC’s digital code to values of R_CNT_.

#### 3.2.2. Quantization Error and Noise

The quantization error in the ROIC is determined by these factors: the clock frequency, which determines the TDC’s resolution; and the regulating voltage and gain of the time signal generator. Equation (4) can be modified to include the quantization error as follows:

(6) $$T_{\text{DIFF}}=\frac{R_{\text{CNT},\text{Real}}\times C_{\text{CNT},\text{Real}}\times \Delta {V^{\prime }}_{\text{CNT}}}{{\text{VDD}}_{\text{CNT}}-{V^{\prime }}_{\text{Ref},\text{CNT}}}-\frac{R_{\text{REF},\text{Real}}\times C_{\text{REF},\text{Real}}\times \Delta {V^{\prime }}_{\text{REF}}}{{\text{VDD}}_{\text{REF}}-{V^{\prime }}_{\text{Ref},\text{REF}}}+Q$$

where the subscript ‘Real’ indicates a real value to be measured after the chip has been fabricated, and Q is the quantization error. Before calibration, the value of R_CNT_ has to be calculated using design values which is indicated with the subscript ‘Designed’, as follows:

(7) $$R_{\text{CNT},\text{Calculated}}=R_{\text{REF},\text{Designed}}+T_{\text{DIFF}}\times \left(\frac{{\text{VDD}}_{\text{CNT}}-V_{\text{Ref},\text{CNT}}}{C_{\text{CNT},\text{Designed}}\times \Delta V_{\text{CNT}}}\right)$$

where the subscript ‘Calculated’ refers to a value measured by the ROIC, V_Ref,CNT_ = V_Ref3_ and ΔV_CNT_ = V_Ref2_ − V_Ref1_. Combining Equations (6) and (7), we obtain:

(8) $$\begin{array}{c} R_{\text{CNT},\text{Calculated}}=R_{\text{REF},\text{Designed}}-E_{1}\;R_{\text{REF},\text{Real}}+E_{2}\;R_{\text{CNT},\text{Real}}+Q\times \;\frac{{\text{VDD}}_{\text{CNT}}-V_{\text{Ref},\text{CNT}}}{C_{\text{CNT},\text{Designed}}\times \Delta V_{\text{CNT}}}\\ {\text{where E}}_{1}=\left(\frac{{\text{VDD}}_{\text{CNT}}-V_{\text{Ref},\text{CNT}}}{{\text{VDD}}_{\text{REF}}-{V^{\prime }}_{\text{Ref},\text{REF}}}\right)\;\left(\frac{C_{\text{REF},\text{Real}}}{C_{\text{CNT},\text{Designed}}}\right)\;\left(\frac{\Delta {V^{\prime }}_{\text{REF}}}{\Delta V_{\text{CNT}}}\right)\\ {\text{and E}}_{2}=\left(\frac{{\text{VDD}}_{\text{CNT}}-V_{\text{Ref},\text{CNT}}}{{\text{VDD}}_{\text{CNT}}-{V^{\prime }}_{\text{Ref},\text{CNT}}}\right)\;\left(\frac{C_{\text{CNT},\text{Real}}}{C_{\text{CNT},\text{Designed}}}\right)\;\left(\frac{\Delta {V^{\prime }}_{\text{CNT}}}{\Delta V_{\text{CNT}}}\right). \end{array}$$

Under perfect calibration, E_1_ = 1, E_2_ = 1, R_REF,Real_ = R_REF,Designed_, and we obtain the simplified equation:

(9) $$R_{\text{CNT},\text{Calculated}}=R_{\text{CNT},\text{Real}}+\gamma Q,\;\text{where}\;\gamma =\frac{{\text{VDD}}_{\text{CNT}}-V_{\text{Ref},\text{CNT}}}{C_{\text{CNT},\text{Designed}}\times \Delta V_{\text{CNT}}}.$$

We can now minimize the quantization error by minimizing γ. The value of VDD_CNT_, V_Ref,CNT_ and ΔV_CNT_ cannot be chosen arbitrarily because of the limited operation range of the ROIC. This leaves C_CNT_ (=C_REF_) as the most useful design parameter. In this case, we should consider that the extent to which we can vary C_CNT_ is also limited, since the value of the capacitor is directly related to the area that it occupies on the chip. And we also need to consider noise when we select the value of C_CNT_.

### 3.3. System Architecture

For measurement, the architecture was implemented onto a CNT sensor board. Its main components are the CNT ROIC, a micro-controller unit (MCU) and a USB interface. The ROIC is mounted on the board after deposition of the CNT on its die. The MCU sets the values and timing of the INIT, RESET, ROWSEL[0:2], COLSEL[0:3] and REFRSEL[0:3] signals. The digital data from the ROIC is sampled and stored by the MCU, which can transfer it to a PC over a USB interface, or directly calculate the resistance of the CNT network. All the components on this board are powered by the USB connection. The form factor of the ROIC section of the board is 35 mm by 87 mm, and the remainder occupies an area of 35 mm by 80 mm. The shape and size of the PCB were determined by the diameter of the measurement system and are not critical parameters.

## 4. Measurement and Results

The NH_3_ sensing experiments were carried out at 100 °C to consider the effect of temperature on sensitivity using the chamber shown in Figure 4. We measured the response of the CNT cells over five cycles, each consisting of two phases: a reaction phase during which the 1,000 ppm NH_3_ is added to the air flowing over the CNT network cells, and a second recovery phase during which CNT network cells are washed by unadulterated air. Operation of the ROIC was started about 15 minutes before the 2nd cycle, to allow time for initialization and stabilization. The duration of each reaction phase was approximately 15 minutes, and the recovery phase lasted around 60 minutes.

**Figure 11.**
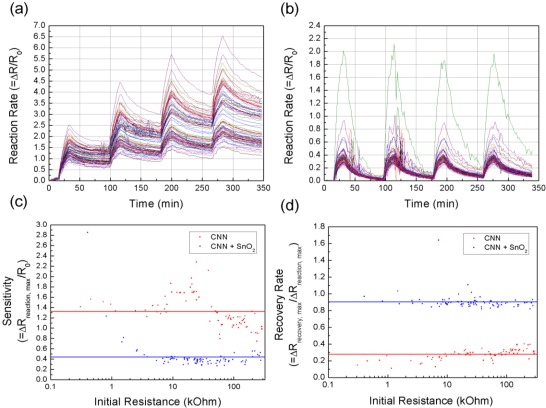
(**a**) Response of the CNT-network cells to 1,000 ppm of NH_3_ at 100 °C: individual reaction rates of 77 cells. (**b**) Response of CNT-SnO_2_ network cells to 1,000 ppm of NH_3_ gas at 100 °C: individual reaction rates of 83 cells. (**c**) Distribution of the sensitivity of the CNT-network cells and the CNT-SnO_2_ network cells. (**d**) Distribution of the recovery rates of the CNT-network cells and the CNT-SnO_2_ network cells.

Figure 11 shows the measured response of the CNT-network cells connected to the ROIC and mounted on the sensor board. The reaction rate ΔR/R_0_ is the change in resistance of each CNT-network cell divided by its initial resistance. Figure 11(a) shows reaction rates of 77 CNT-network cells. The results from the remaining 48 cells were ignored due to excessive instability and noise. Figure 11(a) indicates that most of the CNT network cells operate as expected, and that their resistance increases when NH_3_ is introduced into the reaction chamber. However, there is a nontrivial drift that severely inhibits operational capabilities of this sensor, and suggests the network will have a short lifetime.

Therefore, using the SnO_2_ decorated CNT processes discussed in Section 2, we fabricated modified sensing cells. Figure 11(b) shows the response of these SnO_2_-CNT network cells to exposure of 1,000 ppm of NH_3_ gas at 100 °C. In this case, 83 cells operate correctly with better performance and less deterioration from stage to stage. Figure 11(c,d) shows the distribution of sensitivity and recovery rates, calculated using record-cycle data, across the sensors at 100 °C; normalized to the initial cell resistances. The SnO_2_-CNT network cells have a more uniform distribution than the plain CNT-network cells, the sensitivity of most of the cells lies near the average, except for some low-resistance cells. Although the magnitude of the sensor responses is not constant, the recovery rates of the SnO_2_-CNT-network cells are not only uniform, but also significantly better than the plain CNT. This suggests that ratios between the amounts of gas taken up and then released by the CNT networks are almost constant within a stage, but can vary from stage to stage. Increasing the temperature improves sensitivity, as we would expect because of the higher activation energy. These results show how multi-cell networks not only achieve more reliable results, but also yield more information about the mechanisms operating in their cells.

## 5. Conclusions

We have successfully constructed a sensor in which an array of CNT network cells is combined with an ROIC on a single die. The ROIC combines a counter-based coarse TDC with a DLL-based fine TDC to enhance resolution and reduce power consumption. An analog front-end circuit with simple regulation loops and capacitors generates a time-difference signal which is proportional to the difference between the resistance of a reference resistor and that of the CNT sensor.

We have demonstrated the limitations of bare CNT networks and shown how SnO_2_-CNT networks can improve sensor lifetime and response sensitivity. The implementation of multiple SnO_2_-CNT network cells on a single chip indicates a clear path towards a low-cost and reliable gas sensor for ubiquitous sensor network applications.
